# Bibliometric and meta-analysis on the publication status, research trends and impact inducing factors of JA–SA interactions in plants

**DOI:** 10.3389/fpls.2024.1487434

**Published:** 2024-11-28

**Authors:** Long Jiao, Rongrong Tan, Xun Chen, Hongjuan Wang, Danjuan Huang, Yingxin Mao

**Affiliations:** Key Laboratory of Tea Resources Comprehensive Utilization, Ministry of Agriculture and Rural Affairs, Institute of Fruit and Tea, Hubei Academy of Agricultural Sciences, Wuhan, China

**Keywords:** JA-SA interactions, bibliometric analysis, meta-analysis, plant resistance, phytohormone signaling networks, applied sequence, elicitor concentration, key molecular nodes

## Abstract

Interactions between jasmonic acid (JA) and salicylic acid (SA) pathways in plants are important for regulating metabolite production and resistance functions against environmental stresses. These interactions in plants have mostly been reported to be antagonistic, but also to be synergistic under specific external inducing conditions. At present, publications on plant JA–SA interactions lack a bibliometric analysis. External inducing factors that elicit synergism of JA–SA interactions need to be explored. Here, we use bibliometrics to analyze publications on plant JA–SA interactions over the past three decades, and analyze external inducing factors that influence the quality of JA–SA interactions in plants by meta-analysis. More contributions have been made by authors in China, Netherlands, the United States of America, and Germany than elsewhere. Considerable research has been performed on variation in plant defense mediated by two pathways, the transduction mechanisms of JA–SA signaling crosstalk, and plant hormone signaling networks. Meta-analysis showed that the excitation sequence of the two pathways, and the concentrations of pathway excitors are key factors that affect pathways interactions. The JA and SA pathways tend to be reciprocally antagonistic when elicited simultaneously, whereas JA–SA interactions tend to be synergistic when the two pathways are elicited at different times and the pre-treated inducer is at a lower concentration. The SA pathway is more susceptible to being synergized by the JA pathway. Key molecular nodes identified in the JA–SA signaling interaction in model plants, and prospects for future research are discussed.

## 1 Introduction

Jasmonates (JAs) and salicylates (SAs) are two important defense hormones in plants. External stresses such as herbivore feeding and pathogen infection can promote synthesis of JAs and SAs in plants. Internally synthesized or exogenously applied JAs/SAs can elicit signal transduction and defense genes expression related to jasmonic acid (JA) and salicylic acid (SA) pathways, enhance production of resistant metabolites, and improve plant resistance performance (Berens et al., 2016; Robert-Seilaniantz et al., 2011). The resistance functions mediated by JA and SA pathways are highly specific. SA signaling pathway mediating resistance mainly against biotrophic pathogens, hemibiotrophic pathogens and piercing-sucking herbivores. Plants primarily activate JA pathway in response to attacking caused by leaf-chewing herbivores and infection by necrotrophic microbes (Pieterse et al., 2012).

The JA and SA pathways can mutually affect each other from defensive gene expression to resistance performance (Robert-Seilaniantz et al., 2011; Thaler et al., 2012). Upstream, JA–SA interactions are embodied in a complex crosstalk network between molecular players during signaling transduction between the two pathways. The downstream of this crosstalk is the variations in phytohormone levels, enzyme activity, and metabolite production mediated by these two pathways (Proietti et al., 2013, Proietti et al., 2018; Schweiger et al., 2014). The series of top-down interactions between these pathways is called JA–SA interactions.

JA–SA pathway interactions have been researched for three decades, since SA was found to inhibit the production of proteinase inhibitors in tobacco cells induced by methyl jasmonate (MeJA) (Rickauer et al., 1992). JA–SA interactions have been studied in more than 40 plant species, and in more than 80% of these studies this relationship was reported as reciprocally antagonistic (Jiao et al., 2022; Rigsby et al., 2019; Thaler et al., 2012). Recent research has demonstrated that the relationship between JA and SA pathways can be influenced by external inducing factors such as the elicitor species and the eliciting conditions of the two pathways, and it can also manifest in mutual synergism, or there can be no effect between them (Moreira et al., 2018; Thaler et al., 2012). For example, when both JA and SA signals are applied at low (typically 10–100 mM) concentrations, there is a transient synergistic enhancement in expression of genes *PDF1.2* (*PLANT DEFENSIN 1.2*) and *PR1* (*PATHOGENESIS RELATED 1*) regulated by both pathways. Antagonism between these pathways is reported at higher (250 mM) concentrations (Mur et al., 2006).

Although considerable progress has been made in understanding plant JA–SA interactions, there still lack a systematic analysis on the publication information, variation trend of research hotspots, and external inducing factors affecting the interaction between two pathways. Combing the publications on JA–SA interactions helps to better understand the physiological mechanisms and resistance functions of plant responses to environmental stresses. Further exploration of external inducing factors that promote JA–SA synergism is necessary to improve plant resistance and promote the use of exogenous elicitors in both pathways (Jiao et al., 2022). Bibliometrics is a useful tool to map cumulative scientific knowledge and evolutionary nuances in well-established fields by collecting massive amounts of unstructured data and making sense of it in rigorous ways (Donthu et al., 2021). Meta-analysis is a statistical procedure used to analyze combined data to evaluate overall effects, or effect size from different studies, and it can be a major source of concise and current information (Nakagawa et al., 2017). We use bibliometric methods to analyze publication output, countries and organizations in which research was performed, research collaborations, important research directions, hotspots, and research trends in plant JA–SA interactions in past three decades. Inducing factors affecting properties of plant JA–SA interactions were studied using meta-analysis, key molecular nodes in JA–SA interactions that have been identified in model plants are summarized, and research areas warranting further investigation are identified.

## 2 Material and methods

### 2.1 Data search and analysis strategy in bibliometric analysis

Literature published between January 1, 1992, to December 31, 2023, was sourced from the Web of Science, the core collection of which consists of six online indexing databases, using the query formula “(TS=(salicylate) OR TS=(salicylic)) AND (TS=(jasmonate) OR TS=(jasmonic)) AND (TS=(interaction) OR TS=(crosstalk) OR TS = (cross talk) OR TS=(cross-talk)) AND (AB=(plant)).” Initially 2186 publications were identified. After initial screening, the titles and abstracts of each publication were reviewed. Articles unrelated to JA–SA interactions in plants were excluded, as were those not written in English, and without original research data, such as reviews, comments, and letters. After manual screening, 1802 articles were retained for further analysis. Document data were imported into VOSviewer version 1.6.18 (CWTS, Rapenburg, Leiden, Netherlands) for multidimensional and visual presentation of scientific information in a format suitable for bibliometrics. Keywords, research organizations, countries in which research was performed, and the number and citation frequency of publications were analyzed. Networks of co-occurrence of keywords and co-authorship of organizations and countries were created.

### 2.2 Article screening for meta-analysis

Study inclusion criteria were: (1) the metabolism or resistance performance of plants mediated by JA and SA pathways had to be reported; (2) research had to involve elicitation of the two pathways by exogenous chemical elicitors in different sequences of application or at different concentrations; and (3) a measure of the treatment level means and variability (i.e., variance, standard error, or standard deviation) as the sample size had to be provided in either the text, figures, tables, or appendices. Case studies that concentrated on plant genetic or transcriptional levels of the two pathways, and those in which the JA and SA pathways were induced by biological or abiotic stresses such as herbivore feeding, pathogen infection, light exposure, drought or flooding, were similarly excluded. After screening, 18 studies remained and were included in our meta-analysis. **Supplementary Figure S1** shows the procedures of article searching and screening.

### 2.3 Effect size calculation

Meta-analysis used the log response ratio to quantify effect size across experiments. The log response ratio is defined as ln (*X_d_*/*X_s_*), where *X_d_* is the mean value of effect indicators when both JA and SA pathways were activated, and *X_s_* is the mean value when only the JA or the SA pathway was activated. For negative effect indicators of these pathways, such as herbivore growth rate, population, and pathogen infection rate, values of *X_d_* and *X_s_* were swapped. To accommodate for uncertainty in effect size across experiments, the sample variance for the log response ratio was calculated (Hedges and Curtis, 1999).

The meta-analysis was structured as a multilevel random effects model to account for multiple sources of random variation and to test for hypothesized predictors of effect size (Karban et al., 2014). We chose to test each predictor using univariate models in which only the focal predictor was included, along with the sampling error and random effects terms. In these univariate models, we excluded those studies for which a log response ratio could not be calculated. Studies with continuous independent factors or no replication were also excluded from further analyses. Models were fit using the Bayesian mixed models package MCMC glmm in R version 2.15.2 (Hadfield, 2010). For binary predictors, we report 95% highest posterior density (HPD) credible intervals for the difference in effect size between categories. For categorical predictors with more than two levels, we quantified the variation explained by a factor using the standard deviation across factor levels (Karban et al., 2014), and report the 95% HPD credible interval for this standard deviation.

### 2.4 Statistical analyses

Slopes of numbers of papers published over time in three stages are obtained by linear fitting of publication numbers using GraphPad Prism 10.2.0 (GraphPad Software, Boston, MA, USA). Cluster and neural network analyses of the countries in which research was performed, the organizations involved in the research, and research hotspots were completed using VOSviewer version 1.6.18 (CWTS, Rapenburg, Leiden, Netherlands). Calculation of publication bias and funnel plots in meta-analysis were completed by Review Manager 5.4.1 (The Cochrane Collaboration). Before comparing for effect size, data were lg (X+0.00001) transformed to normalize their distribution and homogenize variances. Independent samples *t*-tests were performed using SPSS 26 software (IBM, Armonk, NY, USA) to compare differences in effect size between JA and SA pathways.

## 3 Results

### 3.1 Annual publication output of research on JA–SA interactions in plants

**Figure 1:**
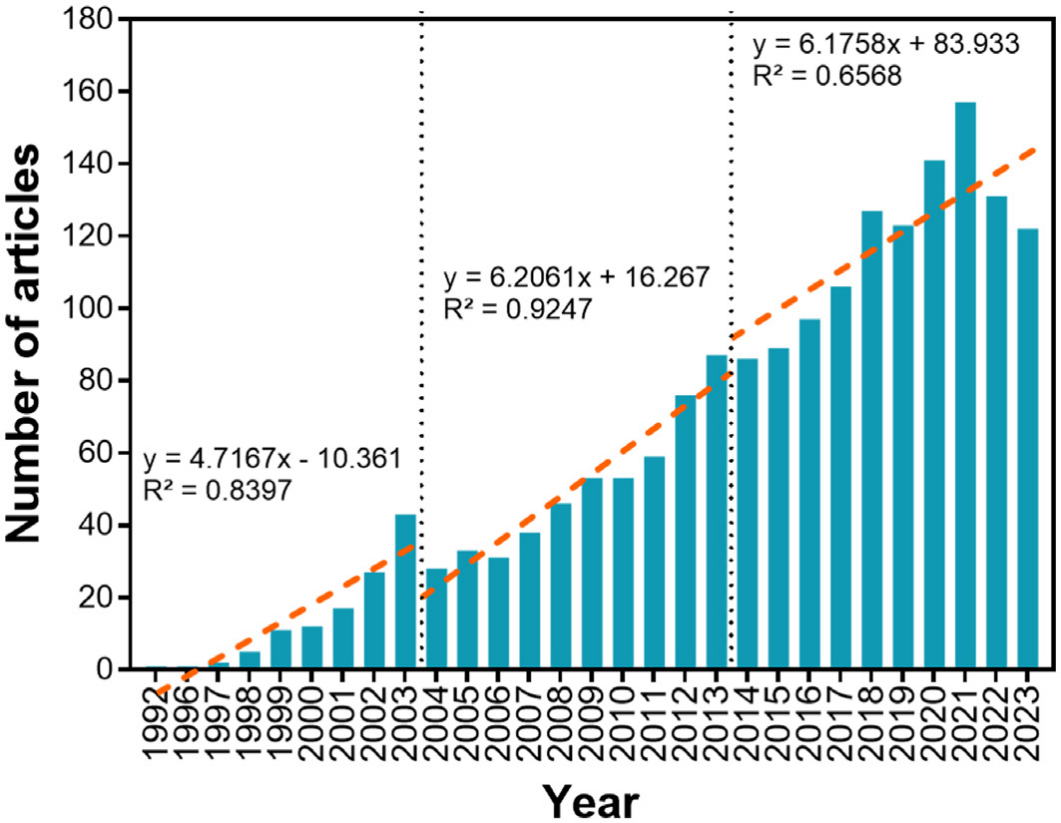
Annual variation in numbers of publications involving plant JA–SA interactions. Red dashed lines represent variation in numbers of publications over time, and subdivides the bibliographic period into three developmental stages. Slopes for research amounts published in each stage are obtained by linear fitting of publication numbers.

The number of publications published annually from 1992 to 2023 is presented in **Figure 1**. Since 1992, the number of publications has increased from 1 annually (1992) to 122 annually (2023), at an overall annual growth rate of 17.37%. Two dashed lines divide the research process into three stages: from 1992–2003 (stage 1), 2004–2013 (stage 2), and 2014–2023 (stage 3). Numbers of publications in stages 1, 2, and 3 account for 6.6%, 27.97%, and 65.43% of all published research (1802 in total), respectively. Slopes of numbers of publications for each stage are obtained by linear fitting publication numbers (stage 1, y’ = 4.7167; stage 2, y’ = 6.2061; stage 3, y’ = 6.1758). The average growth rate in number of research publications in stage 2 was 1.32× that of stage 1, and 1.01× that of stage 3.

### 3.2 Countries, organizations, and collaborations

**Figure 2:**
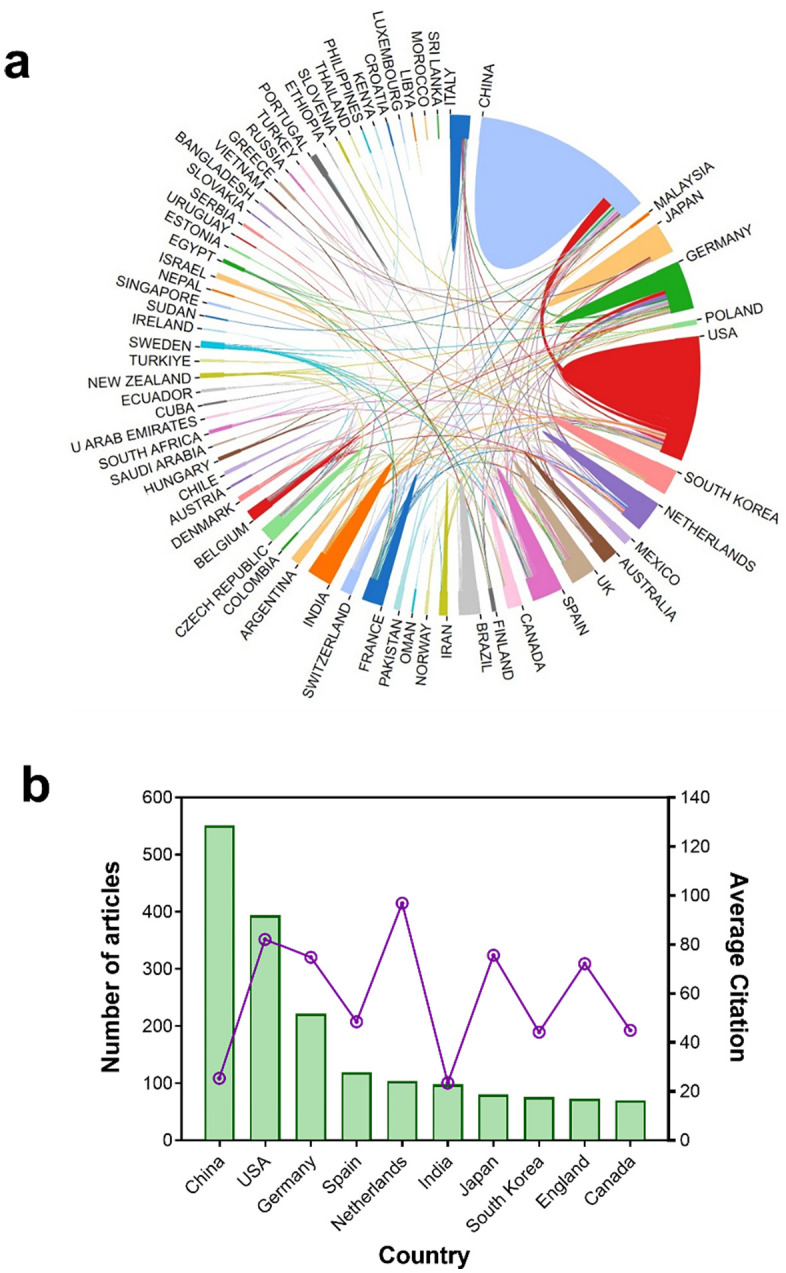
Publication output and collaboration between countries. **(A)** Total numbers of publications and their collaborative relationships among 64 countries. Sector area represents the total number of publications per country. Line thickness between countries represents the extent of collaboration between them. Turkey" (before 2022) and "Türkiye" (after 2022) are the two names for the same country in different periods. **(B)** The top 10 countries (in total numbers of publications) on plant JA–SA interactions and their average citation. The bar chart shows the total number of publications, and the line chart shows the average citation of publications per country.

In total, 64 countries participated in research on JA–SA interactions in plants (**Figure 2A**). The 10 countries with the most publications (and their citations) are depicted in **Figure 2B**. China ranks first (551 articles, 30.58% of all research), followed by the USA (394 articles, 21.86%), and Germany (222 articles, 12.32%). From the average citation of publications, the Netherlands ranks first with 96.83 citations per paper, followed by the USA (82.18 citations), Japan (75.58 citations), Germany (74.81 citations), and England (72.15 citations).

**Figure 3:**
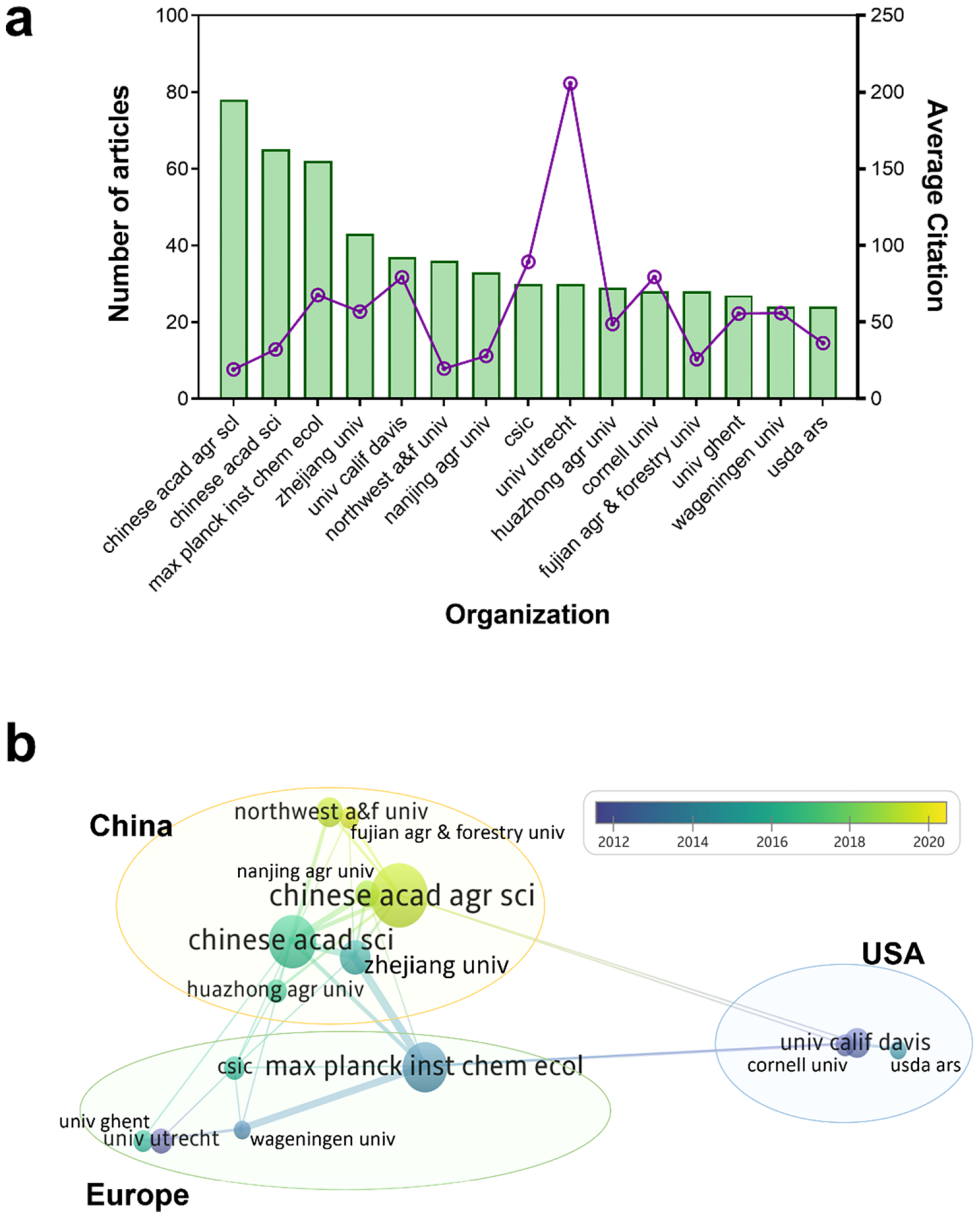
The top 15 organizations in terms of total number of publications on plant JA–SA interactions, and their collaboration. **(A)** The total numbers and average citation of publications published by these organizations. The bar chart shows the total number of publications, and the line chart shows the average citation of publications per organization. **(B)** The collaboration between these organizations. Organization color depicts the year of publication; sphere size represents the total number of publications per organization; and line thickness between organizations represents the extent of collaboration. The organizations included in yellow, green and blue circles are, respectively, located in China, Europe and USA. Chinese acad agr sci, Chinese Academy Of Agricultural Sciences (China); Chinese acad sci, Chinese Academy of Sciences (China); Max planck inst chem ecol, Max Planck Institute for Chemical Ecology (Germany); Zhejiang univ, Zhejiang University (China); Univ calif davis, University of California, Davis (USA); Northwest a&f univ, Northwest A&F University (China); Nanjing agr univ, Nanjing Agricultural University (China); Csic, Spanish National Research Council (Spain); Univ utrecht, Utrecht University (Netherlands); Huazhong agr univ, Huazhong Agricultural University (China); Cornell univ, Cornell University (USA); Fujian agr & forestry univ, Fujian Agriculture and Forestry University (China); Univ ghent, Ghent University (Belgium); Wageningen univ, Wageningen University & Research (Netherlands); Usda ars, United States Department of Agriculture-Agricultural Research Service (USA).

Research involved 1662 organizations, of which the top 15 (with the most publications) are illustrated in **Figure 3**. The top three are of these are the Chinese Academy of Agricultural Sciences (79 articles, 4.38%), Chinese Academy of Sciences (66 papers, 3.66%), and Max Planck Institute for Chemical Ecology (62 articles, 3.44%) (**Figure 3A**). During the development stage, the University of California, Davis, Utrecht University, and Cornell University first published research on plant JA–SA interactions in the 1990s. The Max Planck Institute for Chemical Ecology and Wageningen University & Research published most research on plant JA–SA interactions in stage 2, and the Chinese Academy of Agricultural Sciences and the Chinese Academy of Sciences published most research in stage 3 (**Figure 3B**).

Collaboration between countries and organizations indicates regional characteristics of research on JA–SA interactions in plants. China, with the most publications, has established domestic collaborators, and international collaborations with organizations in Europe (Germany and Netherlands) (**Figure 3B**). Although Spain also has many publications, collaborations between the Spanish National Research Council and other organizations are few (**Figure 3**). USA organizations (the University of California, Davis, and Cornell University) were more inclined to collaborate intra-regionally, but publications were typically of high quality (**Figure 3**). In brief, research on JA–SA interactions in plants becomes more international over time.

### 3.3 Crucial research directions, hotspots, and research trends

**Figure 4:**
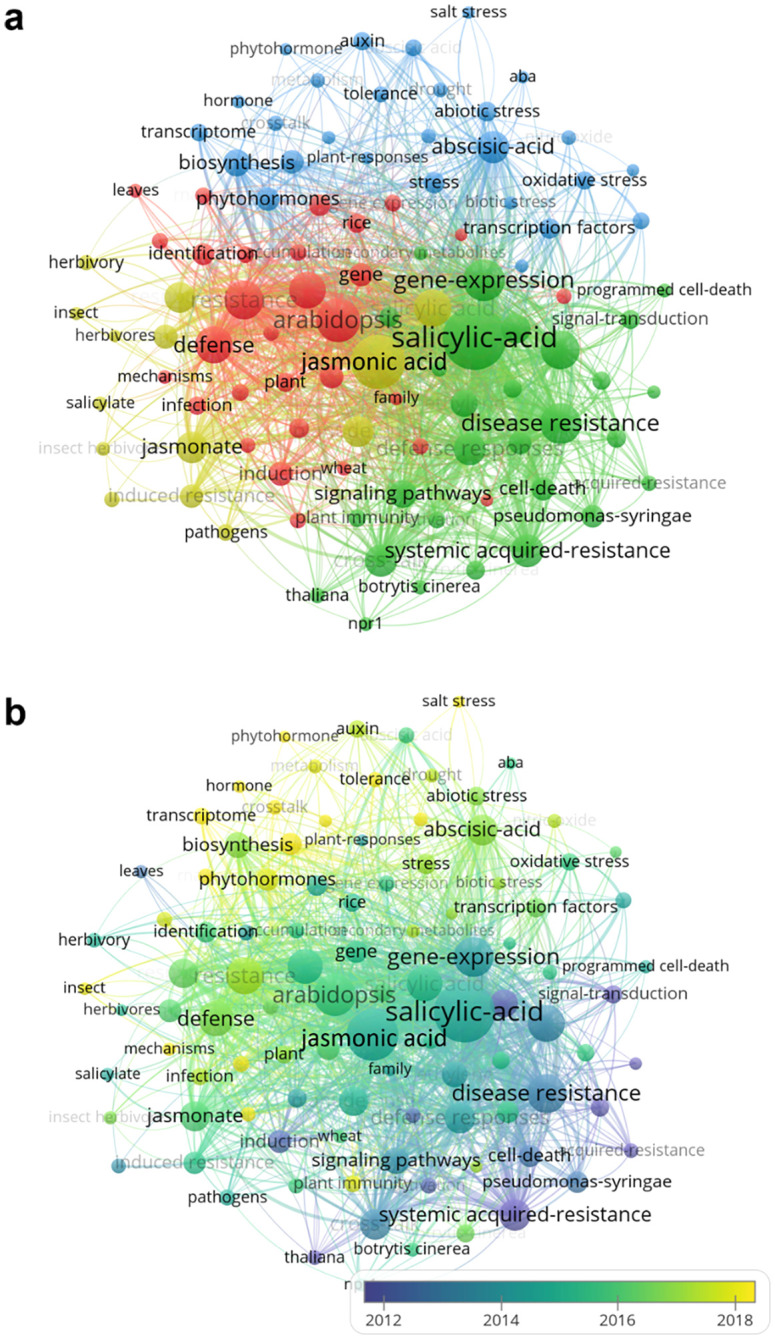
Variation in research hotspots involving plant JA–SA interactions. **(A)** Hotspots based on keywords that appeared more than 30 times. Circles in different colors represent keyword category. **(B)** Variation of hotspots from 1992–2023. Keyword color indicates the year (data based on average values) that keywords appeared.

The frequency of and variation in keywords enable identification of research hotspots. After replacing synonyms, 7076 keywords were extracted from selected articles. The top 100 keywords (cited more than 30 times) are presented in **Figure 4**. Circle size represents keyword frequency, and color, the cluster to which they are allocated, as determined by VOSviewer (**Figure 4A**): cluster I (green) mainly includes salicylic acid, disease resistance, systemic acquired-resistance, NPR1 (NON-EXPRESSOR OF PATHOGENESIS-RELATED GENES 1), and cell death; cluster II (yellow) keywords focus on the jasmonate signaling pathway in plant cells, and are commonly related to herbivore feeding and induced resistance; cluster III keywords (red) include *Arabidopsis*, gene, identification and mechanisms; and cluster IV keywords (blue) are words such as abscisic-acid, auxin, and abiotic stress.

Variation in keywords over time is depicted in **Figure 4B**. A color change from purple to yellow represents the appearance of new keywords over time. Cluster I (close to purple) includes keywords such as systemic acquired-resistance, signal-transduction, defense response, induction, and activation. Keywords in blue such as jasmonic acid, salicylic-acid, disease resistance, herbivory, signaling pathways, and gene-expression, group into cluster II. Clusters III and IV (between green and yellow) contain the most popular keywords between 2014 and 2023, among which the frequencies of “abscisic-acid,” “auxin,” “abiotic stress,” “transcriptome,” and “metabolism” increased significantly.

Combining the timeline in **Figure 3B** and **Figure 4B** for analysis, those publications focused on keywords in cluster I were mainly made by the USA and European organizations (University of California, Davis, Cornell University and Utrecht University). Keywords in cluster II were mostly concentrated by those organizations in Europe, China and USA (Max Planck Institute for Chemical Ecology, Wageningen University & Research, Zhejiang University and United States Department of Agriculture-Agricultural Research Service). Part of Chinese and European organizations (Chinese Academy of Sciences, Huazhong Agricultural University, Spanish National Research Council and Ghent University) mainly focused on the research related to the keywords in cluster III. Keywords in cluster IV were primarily concentrated by Chinese organizations, such as Chinese Academy of Agricultural Sciences, Nanjing Agricultural University, Northwest A&F University and Fujian Agriculture and Forestry University.

### 3.4 External inducing factors that influence the properties of JA–SA interactions in plants

**Figure 5:**
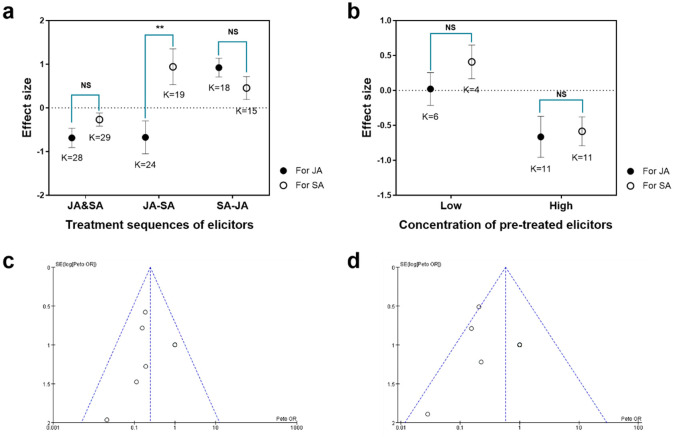
Meta-analysis of external inducing factors that influence the quality of JA–SA interactions in plants. **(A)** Univariate tests of elicitor application sequence that influence effect size (log response ratios) of JA–SA interactions. JA&SA, JA and SA pathways elicited simultaneously; JA-SA, JA pathway pre-elicited and SA pathway post-elicited; SA-JA, SA pathway pre-elicited and JA pathway post-elicited.. **(B)** Univariate tests of concentration of applied elicitors that influence effect size (log response ratios) of JA–SA interactions. Negative values indicate that a pathway is antagonized by another one; positive values indicate that a pathway is synergized by another one. Asterisks indicate significant differences in effect size between pathways (independent samples *t*-test; ^∗∗^ *P* < 0.01, NS means not significant). Low or high concentrations were, respectively, defined as those concentrations less or greater than the middle concentrations used in cases. K=number of research cases in publications. **(C)** Funnel plot for studies on JA–SA interactions in which two pathways were elicited by elicitors in different application sequences. **(D)** Funnel plot for studies on JA–SA interactions in which the two pathways were elicited by elicitors at different concentrations.

A total of 18 publications in which the JA and SA pathways were elicited by different chemical inducers in different sequences of application were screened. The JA and SA pathways were reciprocally antagonistic when the two pathways were elicited simultaneously (JA&SA; *t*= −1.571, *df* = 55, *P* = 0.122; **Figure 5A**). In contrast, JA–SA interactions were reciprocally synergistic when the SA pathway was pre-elicited and the JA pathway was post-elicited (SA-JA; *t=* 1.385, *df*= 41, *P* = 0.174; **Figure 5A**). When the JA pathway was pre-elicited and the SA pathway was post-elicited, the JA pathway tended to be antagonized whereas the SA pathway was synergized (JA-SA; *t= −2.897, df = 31, P* = 0.007; **Figure 5A**). Seven of these 18 research publications in which the JA and SA pathways were elicited by chemical inducers in different concentrations were screened. Meta-analysis revealed that a pre-treated inducer at a lower concentration significantly promoted JA–SA synergism (*t* = −1.03, *df* = 15, *P* = 0.314; **Figure 5B**); higher concentrations contributed to JA–SA antagonism (*t* = −0.205, *df* = 13, *P* = 0.814; **Figure 5B**). There was no publication bias on these research because sample points were evenly and symmetrically distributed on both sides of the funnel plot (**Figures 5C, D**).

## 4 Discussion

We systematically analyze 1802 publications obtained from the Web of Science using bibliometric and meta-analytical methods. From this we describe the publication status, variation in hotspots, and factors that affect JA–SA interactions in plants. We report developed countries such as the Netherlands, USA, and Germany to play a leading role in research on this subject. Although there are fewer than 100 publications from Japan and England, each was cited more than 70 times. Additionally, developing countries such as China and India have gradually increased their research output in this discipline. Although the average number of citations per paper originating from China and India is only 25.49 and 23.51, respectively, the H index of publications from China is higher, indicating that despite the relatively low average citation frequency, China still published highly cited articles in this field. Those developed countries as the pioneers of research on JA–SA interactions, their studies were initially focused on the JA and SA signal-transduction and plant defense responses mediated by these pathways. As developing countries have become the dominant role in this area, studies at that time focused on the broader molecular and metabolic effects of JA–SA interactions in the whole phytohormone signaling networks.

Plant JA–SA interactions were first discovered after an inverse relationship was found in tobacco between the production of proteinase inhibitors induced by SA and MeJA (Rickauer et al., 1992). In the 1990s, studies concerning this interaction were mainly performed by organizations in the USA, such as the University of California, Davis, and the Cornell University, which mainly focused on variation in plant defense responses induced by exogenous elicitors of these two pathways. These USA organizations were more inclined to collaborate intra-regionally, but their publications were typically of high quality. In the 21st century, organizations in Europe (such as the Utrecht University and Max Planck Institute) became the main research centers. With application of molecular biological techniques, methods have shifted from traditional biological analysis to that of gene expression, and research gradually shifted towards deciphering transduction mechanisms of signaling crosstalk between JA–SA pathways in model plants. Until 2010, Chinese organizations (e.g., the Zhejiang University, and Chinese Academy of Sciences) were the centers of research in this discipline, and frequent collaborations were made with European and American researchers to examine relationships and mechanisms between plant resistance to herbivore borne diseases (mediated by the SA pathway) and herbivore feeding (mediated by the JA pathway). Between 2014 and 2023 research began to examine hormone signaling networks in plants, with hotspots shifting to relationships among the two pathways and other hormone pathways, such as the abscisic-acid, auxin, and ethylene signaling pathways. Variation in plant defense response mediated by these two pathways, transduction mechanisms of JA–SA signaling crosstalk, and plant hormone-signaling networks are the three main areas of research that have been intensively investigated, realizing the relevant research is gradually transforming from the phenotypic level to the genetic and molecular level. At present, the latest research focused on the functions and mechanisms of JA–SA interactions in plant response to global climate change, environmental pollution and nutritional deficiency (Montejano-Ramírez and Valencia-Cantero, 2023; Shaffique et al., 2023; Zhang et al., 2024). Moreover, a strategy to synthesize dual SA and JA responsive promoters by combining SA and JA responsive cis elements based on the interaction between their cognate trans- acting factors were developed and can be used for the design of plant biotically or abiotically inducible systems (Li et al., 2023).

**Figure 6:**
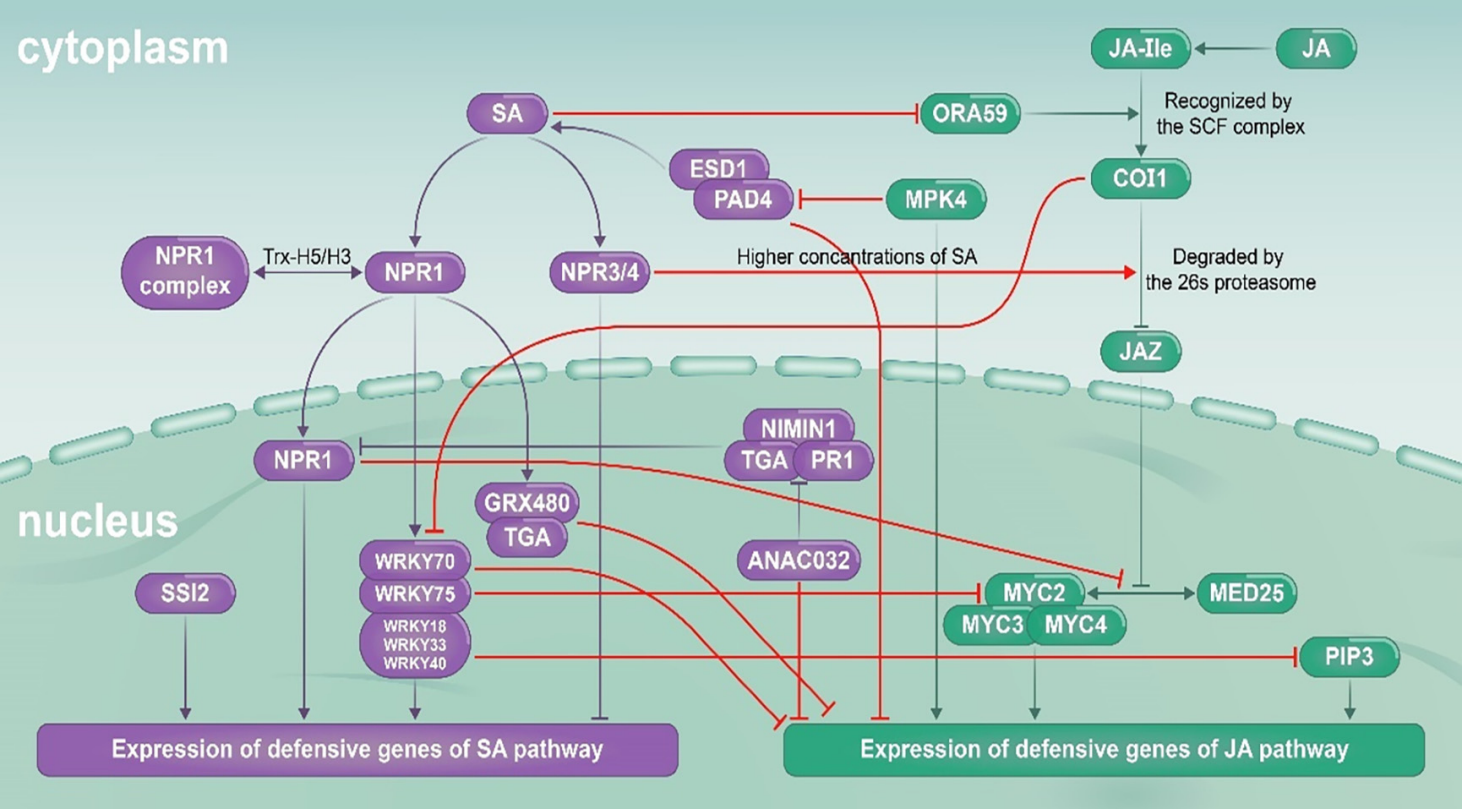
JA (green) and SA (purple) signaling pathways, and interactions between them (red). Arrows indicate synergy, and termination symbols indicate antagonism. COI1, CORONATIN INSENSITIVE 1; EDS1, ENHANCED DISEASE SUPPRESSIBLE 1; GRX480, GLUTAREDOXIN 480; JA, jasmonic acid; JA-Ile, jasmonic acid-isoleucine; JAZ, JASMONATE ZIM-DOMAIN; MED25, MEDIATOR 25; MPK4, MITOGEN-ACTIVATED PROTEIN KINASE 4; MYC, MYELOCYTOMATOSIS; NIMIN1, NIM1-INTERACTING 1; NPR, NON-EXPRESSOR OF PATHOGENESIS-RELATED GENES; TGA, TGACG-MOTIF BINDING PROTEIN; ORA59, OCTADECANOID-RESPONSIVE *Arabidopsis* AP2/ERF-DOMAIN PROTEIN 59; PAD4, PHYTOALEXIN DECIENT 4; PIP3, PAMP-INDUCED SECRETED PEPTIDE 3; PR1, PATHOGENESIS RELATED 1; SA, salicylic acid; SCF, Skp1‐Cdc53‐F‐box protein; SSI2, SA INSENSITIVITY 2.

During signal transduction of JA and SA pathways, a wide range of signal crosstalk occurs between molecular nodes. This upstream signal exchange can affect downstream metabolism production and ecological functions mediated by these pathways (Jiao et al., 2022; Patt et al., 2018; Schweiger et al., 2014). In the past years, our understanding of the molecular mechanisms underlying JA–SA signaling crosstalk in model plants has improved greatly. The key molecular nodes in JA–SA signal crosstalk identified in model plants are summarized in **Table 1**. The model of signal crosstalk between JA and SA pathways is showed in **Figure 6**. In general, it is believed that the SA pathway plays a dominant role in JA–SA interactions. Multiple nodes such as NPR1, TGA (TGACG-MOTIF BINDING PROTEIN), EDS1 (ENHANCED DISEASE SUPPRESSIBLE 1), WRKY, and GRX480 (GLUTAREDOXIN 480) in the SA pathway are involved in inhibition of the JA signaling pathway (Van der Does et al., 2013; Meyer, 2008; Ndamukong et al., 2007; Nomoto et al., 2021; Zander et al., 2012). NPR1 is a master regulator of the defense transcriptome induced by SA and regulate the suppression of JA signaling in the cytosol (Dong, 2004). NPR1 promotes the transcription of WRKY70 which binds to the promoter region of PR1 and induces SA related defenses and represses the JA signaling response (Shim et al., 2013). The NPR polymers are monomerized by thioredoxin and the monomers are transported to the nucleus. The NPR monomers bind to TGAs and regulate the expression of *PR1* genes (Fu et al., 2012). In addition, the wild type NPR1 also negatively regulates SA production during the herbivore attacking, promotes JA related defenses against herbivores by inhibiting the JA–SA interaction. At present, the cryo-electron microscopy and crystal structures of *Arabidopsis* NPR1 and its complex with the transcription factor TGA3 has been reported, which may promote the understanding of its mechanisms in activating plant immunity and regulating the JA–SA interaction (Kumar et al., 2022). Relatively few reports have examined inhibition of SA pathway by JA pathway. In *Arabidopsis*, MPK4 (MITOGEN ACTIVATED PROTEIN KINASE 4) can positively regulate expression of genes such as *PDF1.2* in the JA pathway and inhibit EDS1/PAD4 (PHYTOALEXIN DECIENT 4) mediated SA accumulation in the SA pathway (Brodersen et al., 2006; Wiermer et al., 2005); and COI1 (CORONATIN INSENSITIVE 1) can inhibit expression of PR genes regulated by the SA pathway by inhibiting WRKY70 (Ren et al., 2008).

**Table 1:** Key molecular nodes of JA–SA signal crosstalk identified in model plants.

Interaction nodes (JA pathway)	Interaction nodes (SA pathway)	Interaction mechanism	Interaction quality	References
MYCs and MED25	NPR1	NPR1 promotes the transcription of *WRKY70* which binds to the promoter region of PR1 and induces SA related defenses and represses the JA signaling response. NPR1 inhibited the interaction between MYCs and MED25 in JA pathway. Expression of JA-responsive genes *LOX2*, *PDF1.2* and *VSP2* was enhanced in npr1 mutant than wild type.	Antagonism	Koornneef (2008) ^1^, Nomoto et al. (2021) ^1^, Spoel et al. (2003) ^2^ Shim et al. (2013) ^3^
	OXIDATION-RELATED ZINC FINGER1 (OZF1)	SA can significantly promote MeJA-induced *PDF1.2* expression in the absence of OZF1.	Antagonism	Singh and Nandi (2022) ^4^
PIP3	WRKY18, WRKY33, and WRKY40	WRKY18, WRKY33, and WRKY40 cooperatively act as repressors for PIP3.	Antagonism	Najafi et al. (2020) ^5^
MYCs	WRKY75	WRKY75 as an essential factor controlling SA/JA cross talk by diminishing MYC protein levels.	Antagonism	Schmiesing and Emonet (2016) ^6^
NIMIN1	ANAC032	ANAC032 activates SA signaling by repressing NIMIN1, and reduces expression of *MYC2* and *PDF1.2*.	Antagonism	Allu et al. (2016) ^7^
	GRX480 and TGA	Suppression of *PDF1.2* and *VSP2* by GRX480 depends on the presence of TGA factors.	Antagonism	Koornneef (2008) ^1^, Ndamukong et al. (2007) ^8^
MPK4	EDS1/PAD4	MPK4 negatively regulates EDS1/PAD4, and positively regulates expression of *PDF1.2*. EDS1/PAD4 are important activators of SA signaling and mediate antagonism between JA and ET defense response pathways.	Antagonism	Brodersen et al. (2006) ^9^, Wiermer et al. (2005) ^10^
SCF^COI1^ JAZ complex		The SA pathway inhibits JA signaling downstream of the SCF^COI1^ JAZ complex by targeting GCC-box motifs in JA-responsive promoters via a negative effect on the transcriptional activator ORA59.	Antagonism	Van der Does et al. (2013) ^1^
SSI2		The recessive ssi2 mutation confers constitutive *PR* gene expression. The expression of defensin gene *PDF1.2* regulated by the JA signaling pathway was impaired in ssi2 plants.	Antagonism	Kachroo et al. (2001) ^11^, Shah et al. (2001) ^12^
COI1, JAZs	NPR3, NPR4	Early induction of JA-responsive genes and *de novo* JA synthesis following SA accumulation is activated through the SA receptors NPR3 and NPR4, instead of the JA receptor COI1. NPR3 and NPR4 may mediate this effect by promoting degradation of the JA transcriptional repressor JAZs.	Synergism	Liu et al. (2016) ^13^

Abbreviation notes see **Figure 6**. ^1^Utrecht University, Netherlands; ^2^Seoul National University, Korea; ^3^Nagoya University, Japan; ^4^Jawaharlal Nehru University, India; ^5^Norwegian University of Science and Technology, Norway; ^6^University of Lausanne, Switzerland; ^7^Max Planck Institute of Molecular Plant Physiology, Germany; ^8^Gottingen University, Germany; ^9^Copenhagen University, Denmark; ^10^Max Planck Institute for Plant Breeding Research, Germany; ^11^The State University of New Jersey, USA; ^12^Boyce Thompson Institute for Plant Research, USA; ^13^Duke University, USA. The affiliation of research is based on the first author.

Although JA–SA interactions have mostly been reported as reciprocally antagonistic, increasing evidence suggests that these two pathways can synergize with each other (Jiao et al., 2022; Moreira et al., 2018; Thaler et al., 2012). For example, in *Arabidopsis*, when SA accumulates at higher concentrations, the SA receptor NPR3/NPR4 can replace the JA receptor COI1 to promote degradation of JAZ (JASMONATE ZIM-DOMAIN) protein, and further promote expression of the JA pathway (Liu et al., 2016). We use meta-analysis to analyze research on those external factors that affect the quality of JA–SA interactions. The excitation sequence of the two pathways, and the concentrations of the two pathway excitors are key factors that affect the interaction between them. The JA and SA pathways tend to be reciprocally antagonistic when they are elicited simultaneously. In contrast, JA–SA interactions tend to be synergistic when two pathways are elicited at different times, with the pre-treated inducer at a lower concentration. The SA pathway is more susceptible to being synergized by the JA pathway. Most studies have used simultaneous activation to examine the interaction between pathways, leading to many reports on JA–SA antagonism. When the two pathways are excited at intervals and the concentration of the first applied exciton is lower, there is a synergistic interaction between them. Plant-mediated effects of initial attackers on performance of subsequent attackers were also studied by meta-analysis to test the influences of the biological inducers on the JA–SA interactions (Moreira et al., 2018). During the JA–SA interactions mediated by herbivores or pathogens attacking, effects on subsequent elicited pathway are present when the initial attacker is a herbivore but not when the initial inducer is a pathogen, and interactions involving JA-associated initial herbivores are stronger or more consistent when both attackers feed on the same plant part (Moreira et al., 2018).

Antagonism of the JA pathway to the SA pathway in simultaneous elicitation is likely to be related to resource competition in the plant. When plants face multiple stresses with limited resources, they usually prefer to express the prior elicited pathway but inhibit the other one to deal with the primary stress (Rayapuram and Baldwin, 2007; Züst and Agrawal, 2017). However, less is known about synergism between these two pathways. The defense priming of plants can be induced by exogenous phytohormones at lower concentrations (Erb et al., 2015; Niinemets, 2010; Vos et al., 2013; Ye et al., 2013). When primed, the defense activation of plants in response to biotic and abiotic stresses is faster and stronger (Conrath et al., 2015; Mauch-Mani et al., 2017). Further research is needed to determine if lower JA concentrations can enhance the plant’s SA pathway response by stimulating a defense-priming reaction.

Understanding synergism between JA–SA pathways is important for improving plant resistant performance, and to promote development and application of chemical elicitors. For example, volatiles released by SA-induced tea plants can repel the tea gray geometrid *Ectropis grisescens*, whereas those induced by JA pathway can attract the parasitic wasp *Apanteles* sp. Compared with induction by either pathway, tea plants induced by JA–SA synergism are equipped with both pest repellent and natural enemy attraction, and the effects of both are stronger (Jiao et al., 2022). This indicates that JA–SA synergism enables plants to induce multifarious and stronger defense responses. Moreover, high costs have limited the large-scale use of JAs in the field. In contrast, the price of the SAs pathway is a fraction (~one thousandth) that of JAs. JA–SA synergism means that SAs may serve as inexpensive enhancers for JAs (Jiao et al., 2023).

We examined publication output, the country in which research was performed, the extent of collaboration between organizations, research trends, hotspots, and inducing factors that affect properties of plant JA–SA interactions. In the past three decades, the key molecular codes and mechanisms underlying the interaction between JA and SA pathways in model plants have been clarified, but issues remain to be addressed. For example, it is believed that the SA pathway takes priority over the JA pathway, but the genetic basis behind this remains unclear (Berens et al., 2016). Furthermore, herbivores can manipulate the JA–SA relationship in plants to cope with their defense performance. Revealing the mechanisms involved in this is important to understand co-evolutionary relationships between plants and insects (Thaler et al., 2012). In addition to JA and SA, the plant hormone signaling network regulates the distribution of primary metabolites between plant growth and defense, called the “trade-off” between the two (Campos et al., 2016; Havko et al., 2016; Züst and Agrawal, 2017). Further study on the network among the various hormone pathways is also required to explain hormone stress resistance mechanisms of plants, and to explore their optimal growth defense balance point.

## Data Availability

The raw data supporting the conclusions of this article will be made available by the authors, without undue reservation.

## References

[B1] Allu A. D. Brotman Y. Xue G. P. Balazadeh S. (2016). Transcription factor ANAC032 modulates JA/SA signalling in response to *Pseudomonas syringae* infection. EMBO Rep. 17, 1578–1589. doi: 10.15252/embr.201642197 27632992 PMC5090710

[B2] Berens M. L. Berry H. M. Mine A. Argueso C. T. Tsuda K. (2016). Evolution of hormone signaling networks in plant defense. Annu. Rev. Phytopathol. 55, 401–425. doi: 10.1146/annurev-phyto-080516-035544 28645231

[B3] Brodersen P. Petersen M. Bjørn Nielsen H. Zhu S. Newman M. A. Shokat K. M.. (2006). *Arabidopsis* MAP kinase 4 regulates salicylic acid- and jasmonic acid/ethylene-dependent responses via EDS1 and PAD4. Plant J. 47, 532–546. doi: 10.1111/j.1365-313X.2006.02806.x 16813576

[B4] Campos M. L. Yoshida Y. Major I. T. Ferreira D. D. O. Weraduwage S. M. Froehlich J. E.. (2016). Rewiring of jasmonate and phytochrome B signalling uncouples plant growth-defense tradeoffs. Nat. Commun. 7, 12570. doi: 10.1038/ncomms12570 27573094 PMC5155487

[B5] Conrath U. Beckers G. J. Langenbach C. J. Jaskiewicz M. R. (2015). Priming for enhanced defense. Annu. Rev. Phytopathol. 53, 97–119. doi: 10.1146/annurev-phyto-080614-120132 26070330

[B6] Dong X. (2004). NPR1, all things considered. Curr. Opin. Plant Biol. 7, 547–552. doi: 10.1016/j.pbi.2004.07.005 15337097

[B7] Donthu N. Kumar S. Mukherjee D. Pandey N. Lim W. M. (2021). How to conduct a bibliometric analysis: An overview and guidelines. J. Bus. Res. 133, 285–296. doi: 10.1016/j.jbusres.2021.04.070

[B8] Erb M. Veyrat N. Robert C. A. Xu H. Frey M. Ton J.. (2015). Indole is an essential herbivore-induced volatile priming signal in maize. Nat. Commun. 6, 6273. doi: 10.1038/ncomms7273 25683900 PMC4339915

[B9] Fu Z. Q. Yan S. Saleh A. Wang W. Ruble J. Oka N.. (2012). Npr3 and npr4 are receptors for the immune signal salicylic acid in plants. Nature 486, 3153. doi: 10.1038/nature11162 PMC3376392 22699612

[B10] Hadfield J. D. (2010). MCMC methods for multi-response generalized linear mixed models: the MCMCglmm R package. J. Stat. Software 33, 1–22. doi: 10.18637/jss.v033.i02

[B11] Havko N. E. Major I. T. Jewell J. B. Attaran E. Browse J. Howe G. A. (2016). Control of carbon assimilation and partitioning by jasmonate: an accounting of growth–defense tradeoffs. Plants 5, 7. doi: 10.3390/plants5010007 27135227 PMC4844420

[B12] Hedges L. V. Curtis G. P. S. (1999). The meta-analysis of response ratios in experimental ecology. Ecology 80, 1150–1156. doi: 10.1890/0012-9658(1999)080[1150:TMAORR]2.0.CO;2

[B13] Jiao L. Bian L. Luo Z. Li Z. Xiu C. Fu N.. (2022). Enhanced volatile emissions and anti-herbivore functions mediated by the synergism between jasmonic acid and salicylic acid pathways in tea plants. Hortic. Res. 9, uhac144. doi: 10.1093/hr/uhac144 36101895 PMC9463459

[B14] Jiao L. Chen X. Tan R. Wang H. Huang D. Mao Y. (2023). Research progress on the interaction between jasmonic acid and salicylic acid signaling pathways in plants. Plant Physiol. J. 59, 1489–1504. doi: 10.13592/j.cnki.ppj.300133

[B15] Kachroo P. Shanklin J. Shah J. Whittle E. J. Klessig D. F. (2001). A fatty acid desaturase modulates the activation of defense signaling pathways in plants. Proc. Natl. Acad. Sci. U.S.A. 98, 9448–9453. doi: 10.1073/pnas.151258398 11481500 PMC55441

[B16] Karban R. Yang L. H. Edwards K. F. (2014). Volatile communication between plants that affects herbivory: a meta-analysis. Ecol. Lett. 17, 44–52. doi: 10.1111/ele.2013.17.issue-1 24165497

[B17] Koornneef A. (2008). Cross-talk in plant defense signaling: Antagonism between salicylate and jasmonate pathways in *Arabidopsis* (Utrecht, Netherlands: Utrecht University).

[B18] Kumar S. Zavaliev R. Wu Q. Zhou Y. Cheng J. Dillard L.. (2022). Structural basis of NPR1 in activating plant immunity. Nature 605, 561–566. doi: 10.1038/s41586-022-04699-w 35545668 PMC9346951

[B19] Li X. Niu G. Fan Y. Liu W. Wu Q. Yu C.. (2023). Synthetic dual hormone-responsive promoters enable engineering of plants with broad-spectrum resistance. Plant Commun. 4, 100596. doi: 10.1016/j.xplc.2023.100596 36998212 PMC10363552

[B20] Liu L. Sonbol F. M. Huot B. Gu Y. Withers J. Mwimba M.. (2016). Salicylic acid receptors activate jasmonic acid signalling through a non-canonical pathway to promote effector-triggered immunity. Nat. Commun. 7, 13099. doi: 10.1038/ncomms13099 27725643 PMC5062614

[B21] Mauch-Mani B. Baccelli I. Luna E. Flors V. (2017). Defense priming: an adaptive part of induced resistance. Annu. Rev. Plant Biol. 68, 485–512. doi: 10.1146/annurev-arplant-042916-041132 28226238

[B22] Meyer A. J. (2008). The integration of glutathione homeostasis and redox signaling. J. Plant Physiol. 165, 1390–1403. doi: 10.1016/j.jplph.2007.10.015 18171593

[B23] Montejano-Ramírez V. Valencia-Cantero E. (2023). Cross-talk between iron deficiency response and defense establishment in plants. Int. J. Mol. Sci. 24, 6236. doi: 10.3390/ijms24076236 37047208 PMC10094134

[B24] Moreira X. Abdala-Roberts L. Castagneyrol B. (2018). Interactions between plant defence signalling pathways: Evidence from bioassays with insect herbivores and plant pathogens. J. Ecol. 106, 2353–2364. doi: 10.1111/jec.2018.106.issue-6

[B25] Mur L. A. Kenton P. Atzorn R. Miersch O. Wasternack C. (2006). The outcomes of concentration-specific interactions between salicylate and jasmonate signaling include synergy, antagonism, and oxidative stress leading to cell death. Plant Physiol. 140, 249–262. doi: 10.1104/pp.105.072348 16377744 PMC1326048

[B26] Najafi J. Brembu T. Vie A. K. Viste R. Winge P. Somssich I. E.. (2020). PAMP-INDUCED SECRETED PEPTIDE 3 modulates immunity in *Arabidopsis* . J. Exp. Bot. 71, 850–864. doi: 10.1093/jxb/erz482 31665431

[B27] Nakagawa S. Noble D. W. Senior A. M. Lagisz M. (2017). Meta-evaluation of meta-analysis: ten appraisal questions for biologists. BMC Biol. 15, 18. doi: 10.1186/s12915-017-0357-7 28257642 PMC5336618

[B28] Ndamukong I. Abdallat A. A. Thurow C. Fode B. Zander M. Weigel R.. (2007). SA-inducible *Arabidopsis* glutaredoxin interacts with TGA factors and suppresses JA-responsive *PDF1.2* transcription. Plant J. 50, 128–139. doi: 10.1111/j.1365-313X.2007.03039.x 17397508

[B29] Niinemets U. (2010). Mild versus severe stress and BVOCs: thresholds, priming and consequences. Trends Plant Sci. 15, 145–153. doi: 10.1016/j.tplants.2009.11.008 20006534

[B30] Nomoto M. Skelly M. J. Itaya T. Mori T. Suzuki T. Matsushita T.. (2021). Suppression of MYC transcription activators by the immune cofactor NPR1 fine-tunes plant immune responses. Cell Rep. 37, 110125. doi: 10.1016/j.celrep.2021.110125 34910911

[B31] Patt J. M. Robbins P. S. Niedz R. McCollum G. Alessandro R. (2018). Exogenous application of the plant signalers methyl jasmonate and salicylic acid induces changes in volatile emissions from citrus foliage and influences the aggregation behavior of Asian citrus psyllid (*Diaphorina citri*), vector of Huanglongbing. PLoS One 13, e0193724. doi: 10.1371/journal.pone.0193724 29596451 PMC5875780

[B32] Pieterse C. M. J. van der Does D. Zamioudis C. Leon-Reyes A. Van Wees S. C. M. (2012). Hormonal modulation of plant immunity. Annu. Rev. Cell Dev. Biol. 28, 489–521. doi: 10.1146/annurev-cellbio-092910-154055 22559264

[B33] Proietti S. Bertini L. Timperio A. M. Caporale C. Caruso C. (2013). Update on crosstalk between salicylic acid and jasmonate in defense signaling: a proteomic approach. IOBC/WPRS Bull. 88, 189–193. 10.1039/c3mb25569g 23624517

[B34] Proietti S. Caarls L. Coolen S. Van Pelt J. A. Scm V. W. Cmj P. (2018). Genome-wide association study reveals novel players in defense hormone crosstalk in *Arabidopsis* . Plant Cell Environ. 41, 2342–2356. doi: 10.1111/pce.v41.10 29852537 PMC6175328

[B35] Rayapuram C. Baldwin I. T. (2007). Increased SA in NPR1-silenced plants antagonizes JA and JA-dependent direct and indirect defenses in herbivore-attacked *Nicotiana attenuata* in nature. Plant J. 52, 700–715. doi: 10.1111/j.1365-313X.2007.03267.x 17850230

[B36] Ren C. M. Zhu Q. Gao B. D. Ke S. Y. Yu W. C. Xie D. X.. (2008). Transcription factor WRKY70 displays important but no indispensable roles in jasmonate and salicylic acid signaling. J. Integr. Plant Biol. 50, 630–637. doi: 10.1111/j.1744-7909.2008.00653.x 18713432

[B37] Rickauer M. Bottin A. Esquerré T. M. T. (1992). Regulation of proteinase inhibitor production in tobacco cells by fungal elicitors, hormonal factors and methyl jasmonate. Plant Physiol. Bioch. 30, 579–584.

[B38] Rigsby C. M. Shoemaker E. E. Mallinger M. M. Orians C. M. Preisser E. L. (2019). Conifer responses to a stylet-feeding invasive herbivore and induction with methyl jasmonate: impact on the expression of induced defences and a native folivore. Agric. For. Entomol. 21, 227–234. doi: 10.1111/afe.2019.21.issue-2

[B39] Robert-Seilaniantz A. Grant M. Jones J. D. (2011). Hormone crosstalk in plant disease and defense: more than just jasmonate-salicylate antagonism. Annu. Rev. Phytopathol. 49, 317–343. doi: 10.1146/annurev-phyto-073009-114447 21663438

[B40] Schmiesing A. Emonet A. (2016). Arabidopsis MYC transcription factors are the target of hormonal salicylic acid/jasmonic acid cross talk in response to *pieris brassicae* egg extract. Plant Physiol. 170, 2432–2443. doi: 10.1104/pp.16.00031 26884488 PMC4825139

[B41] Schweiger R. Heise A. M. Persicke M. Müller C. (2014). Interactions between the jasmonic and salicylic acid pathway modulate the plant metabolome and affect herbivores of different feeding types. Plant Cell Environ. 37, 1574–1585. doi: 10.1111/pce.2014.37.issue-7 24372400

[B42] Shaffique S. Hussain S. Kang S. M. Imran M. Injamum-Ul-Hoque M. Khan M. A.. (2023). Phytohormonal modulation of the drought stress in soybean: outlook, research progress, and cross-talk. Front. Plant Sci. 14, 1237295. doi: 10.3389/fpls.2023.1237295 37929163 PMC10623132

[B43] Shah J. Kachroo P. Nandi A. Klessig D. F. (2001). A recessive mutation in the *Arabidopsis SSI2* gene confers SA- and NPR1-independent expression of *PR* genes and resistance against bacterial and oomycete pathogens. Plant J. 25, 563–574. doi: 10.1046/j.1365-313x.2001.00992.x 11309146

[B44] Shim J. S. Jung C. Lee S. Min K. Lee Y. W. Choi Y.. (2013). Atmyb44 regulates wrky70 expression and modulates antagonistic interaction between salicylic acid and jasmonic acid signaling. Plant J. 73, 483–495. doi: 10.1111/tpj.2013.73.issue-3 23067202

[B45] Singh N. Nandi A. K. (2022). AtOZF1 positively regulates JA signaling and SA-JA cross-talk in *Arabidopsis thaliana* . J. Biosci. 47, 8. doi: 10.1007/s12038-021-00243-6 35092410

[B46] Spoel S. H. Koornneef A. Claessens S. M. Korzelius J. P. Van Pelt J. A. Mueller M. J.. (2003). NPR1 modulates cross-talk between salicylate- and jasmonate-dependent defense pathways through a novel function in the cytosol. Plant Cell 15, 760–770. doi: 10.1105/tpc.009159 12615947 PMC150028

[B47] Thaler J. S. Humphrey P. T. Whiteman N. K. (2012). Evolution of jasmonate and salicylate signal crosstalk. Trends Plant Sci. 17, 260–270. doi: 10.1016/j.tplants.2012.02.010 22498450

[B48] Van der Does D. Leon-Reyes A. Koornneef A. Van Verk M. C. Rodenburg N. Pauwels L.. (2013). Salicylic acid suppresses jasmonic acid signaling downstream of SCFCOI1-JAZ by targeting GCC promoter motifs via transcription factor ORA59. Plant Cell 25, 744–761. doi: 10.1105/tpc.112.108548 23435661 PMC3608790

[B49] Vos I. A. Adriaan V. Schuurink R. C. Watt L. G. Pieterse C. M. J. Van W. S. C. M. (2013). Onset of herbivore-induced resistance in systemic tissue primed for jasmonate-dependent defenses is activated by abscisic acid. Front. Plant Sci. 4, 539. doi: 10.3389/fpls.2013.00539 24416038 PMC3874679

[B50] Wiermer M. Feys B. J. Parker J. E. (2005). Plant immunity: the EDS1 regulatory node. Curr. Opin. Plant Biol. 8, 383–389. doi: 10.1016/j.pbi.2005.05.010 15939664

[B51] Ye M. Song Y. Long J. Wang R. Baerson S. R. Pan Z.. (2013). Priming of jasmonate-mediated antiherbivore defense responses in rice by silicon. Proc. Natl. Acad. Sci. U.S.A. 110, 3631–3639. doi: 10.1073/pnas.1305848110 PMC3780902 24003150

[B52] Zander M. Chen S. Imkampe J. Thurow C. Gatz C. (2012). Repression of the *arabidopsis thaliana* jasmonic acid/ethylene-induced defense pathway by TGA-interacting glutaredoxins depends on their C-terminal ALWL motif. Mol. Plant 5, 831–840. doi: 10.1093/mp/ssr113 22207719

[B53] Zhang Z. Zhang T. Lu L. Qiu S. Huang Z. Wang Y.. (2024). Synergistic interaction between brassinosteroid and jasmonate pathways in rice response to cadmium toxicity. Sci. Total Environ. 954, 176369. doi: 10.1016/j.scitotenv.2024.176369 39299342

[B54] Züst T. Agrawal A. A. (2017). Trade-offs between plant growth and defense against insect herbivory: an emerging mechanistic synthesis. Annu. Rev. Plant Biol. 68, 513–534. doi: 10.1146/annurev-arplant-042916-040856 28142282

