# Supplementary figures and images for: Bibliometric and meta-analysis on the publication status, research trends and impact inducing factors of JA–SA interactions in plants
Source: Front Plant Sci. 2024 Nov 28;15:1487434. doi: 10.3389/fpls.2024.1487434 (PMC11635838; doi:10.3389/fpls.2024.1487434)

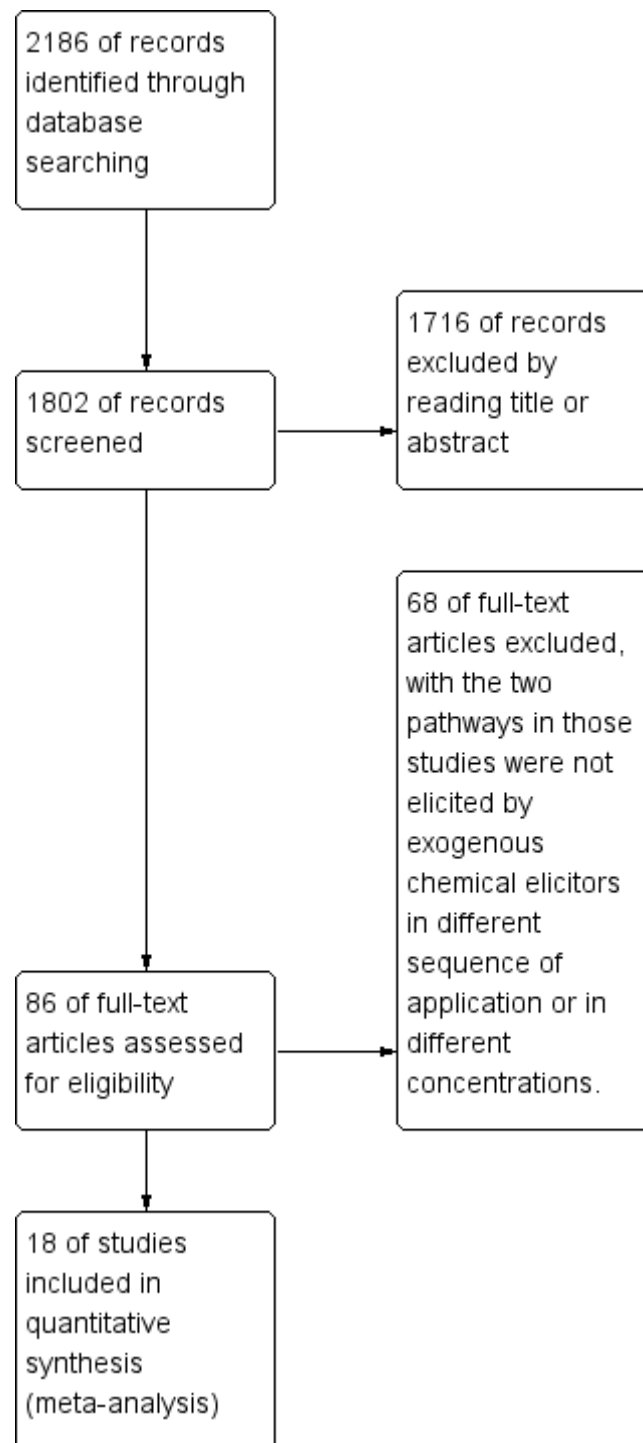

**Figure. S1** PRISMA flow chart revealing numbers of papers retained at each stage.

Supplement: Supplementary file 1 [file Image1.pdf]
